# Colostomy and quality of life after spinal cord injury: systematic review

**DOI:** 10.1002/bjs5.50339

**Published:** 2020-08-27

**Authors:** O. Waddell, A. McCombie, F. Frizelle

**Affiliations:** ^1^ Department of Surgery University of Otago Christchurch 2 Riccarton Avenue, Christchurch Central City Christchurch 8011 New Zealand

## Abstract

**Background:**

Spinal cord injury (SCI) has a significant impact on the quality of life (QoL) of affected patients. The aim of this review was to determine whether colostomy formation improves QoL in patients with SCI.

**Methods:**

The Cochrane Register, MEDLINE, Embase and CINAHL were searched using medical subject headings. The search was extended to the reference lists of identified studies, ClinicalTrials.gov and the WHO International Clinical Trials Registry. All clinical trials that included spinal injury and QoL, time spent on bowel care, and patient satisfaction with stoma were assessed.

**Results:**

A total of 15 studies were found (including 488 patients with a stoma), of which 13 were retrospective cross‐sectional studies and two were case–control studies, one of which was prospective research. Nine of 11 studies focusing on QoL reported that patients' QoL was improved by the stoma, whereas the remaining two studies found no difference. Time spent on bowel care was significantly reduced in all 13 studies that considered this outcome, with patients reducing the average time spent on bowel care from more than 1 h to less than 15 min per day. All 12 studies assessing patient satisfaction with their stoma reported high patient satisfaction.

**Conclusion:**

Stoma formation improves QoL, reduces time spent on bowel care, and increases independence. Stoma is an option that could be discussed and offered to patients with spinal cord injury.

## Graphical abstract

In this systematic review to determine whether colostomy formation improved the quality of life (QoL) in patients with spinal cord injury, outcomes assessed were QoL, time spent on bowel care, and patient satisfaction with the stoma. A total of 15 studies were included; colostomy formation improved QoL and time spent on bowel care, and more than 90 per cent of patients were satisfied with having a stoma.

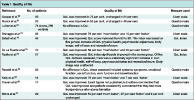

Stoma and spinal cord injury

## Abstract (es)

**Antecedentes:**

La lesión de la médula espinal (*Spinal Cord Injury*, SCI) tiene una clara repercusión en la calidad de vida (*Quality of Life*, QoL) de los pacientes que la sufren. El objetivo de este manuscrito fue determinar si la realización de una colostomía mejora la calidad de vida en pacientes con SCI.

**Métodos:**

Se realizó una búsqueda en los registros Cochrane, MEDLINE, Embase y CINAHL utilizando palabras clave. La búsqueda se extendió a la bibliografía de los estudios identificados, al clinictrials.gov y al Registro Internacional de Ensayos Clínicos de la OMS. Se evaluaron todos los ensayos clínicos que analizaron la lesión espinal y la calidad de vida, el tiempo dedicado al cuidado intestinal y la satisfacción del paciente con el estoma.

**Resultados:**

Se incluyeron 15 estudios (con 488 pacientes) de los que 13 fueron estudios transversales retrospectivos, 2 eran estudios caso‐control y uno era un estudio prospectivo. Nueve de los 11 estudios que analizaron la calidad de vida revelaron que la colostomía mejoraba la calidad de vida de los pacientes, mientras que en los 2 restantes no se encontraron diferencias. El tiempo dedicado al cuidado intestinal se redujo significativamente en los 13 estudios. De promedio, los pacientes redujeron el tiempo dedicado al cuidado intestinal de más de una hora por día a menos de 15 minutos. Los 12 estudios que evaluaron la satisfacción del paciente con la colostomía constataron una alta satisfacción de los pacientes con sus estomas.

**Conclusión:**

La construcción de un estoma mejora la calidad de vida, reduce el tiempo dedicado al cuidado intestinal y aumenta la independencia. El estoma es una opción que podría discutirse y ser ofrecida a pacientes con lesión de la médula espinal.

## Introduction

Spinal cord injury (SCI) is a life‐changing injury. The WHO estimates the global incidence of SCI to be 40–80 new cases per million population per year, which equates to between 250 000 and 500 000 new cases internationally each year^1^. In New Zealand, approximately 80–130 people are diagnosed with SCI every year^2^. Young men are by far the most likely to be injured, with the most common cause being motor vehicle accidents followed by falls^2^. Fortunately, with good medical care and support, life expectancy is comparable to that in the able‐bodied population^3^, ^4^, ^5^.

Despite these advances in medical care, patients with SCI experience significant changes in functional status. Far more than just reducing mobility, SCI will affect every aspect of a person's life, including physical, psychosocial, body image, independence, work life, sexuality and recreation/leisure. All of these factors can result in a decreased quality of life (QoL). Issues with bowel care rank highly in terms of their impact on patients' lives^6^. The majority of patients cite bowel control as their greatest functional loss after loss of mobility^7^, with many ranking it worse than both bladder and sexual dysfunction^8^, ^9^. Nearly half of patients state that bowel dysfunction has a major negative impact on their social life^10^, resulting in a decreased QoL^11^, ^12^.

There are many interventions, both conservative and surgical, that can help to improve bowel dysfunction, but dissatisfaction with bowel care remains high. A colostomy or stoma has traditionally been left as a ‘last resort’ option for patients in whom conservative measures have failed, but for many patients a stoma may be a far more effective method of improving bowel dysfunction and enable them to gain control and independence over this aspect of their lives. This systematic review aims to examine the evidence of the impact of stoma formation on bowel function and overall QoL.

Although many studies have been performed on SCI‐related stomas, a systematic review of the literature is lacking. This paper aimed to perform a systematic review of the literature assessing the impact of stoma formation on bowel function and overall QoL in patients with SCI. The three outcomes extracted for review are impact on QoL, time spent on bowel care, and patient satisfaction with their stomas.

## Methods

Studies published to 31 January 2020 and assessing an adult population (age 18 years or above) with an acquired SCI who had been given a stoma were included. Any indication for the stoma formation and any type of intestinal stoma were included. Prospective and retrospective studies with or without a control group were included only if they assessed the effect on stoma formation on one or more of the three outcomes (QoL, time spent on bowel care, and satisfaction with stoma formation). Studies needed to be published in English in a peer‐reviewed journal; conference abstracts were not included.

A literature search of the Cochrane Central Register of Controlled Trials, MEDLINE, Embase and the Cumulative Index to Nursing and Allied Health Literature (CINAHL) was performed using medical subject headings. Reference lists of identified studies, ClinicalTrials.gov and the WHO International Clinical Trials Registry were also searched. All clinical trials that included spinal injury and QoL, time spent on bowel care, and patient satisfaction with stoma were assessed, with restrictions to human adults (age 18 years and above) and English language, and use of the PRISMA‐P checklist. MEDLINE, Embase and PubMed were searched on 23 and 30 March 2019, and again on 31 January 2020 for the terms ‘Spinal Cord Injury’ and ‘Colostomy’ or ‘Stoma’. Studies were screened by title and then abstract by two independent reviewers, and duplicates were removed.

The studies included were assessed for quality using a study quality assessment tool designed by the National Heart, Lung and Blood institute of the USA^13^.

### Data synthesis

As outcomes were predominantly qualitative, a quantitative meta‐analysis was not performed. Time spent on bowel care could be synthesized, but the exact number for this outcome is less relevant than the presence of a significant reduction in the time spent.

## Results

**Fig. 1:**
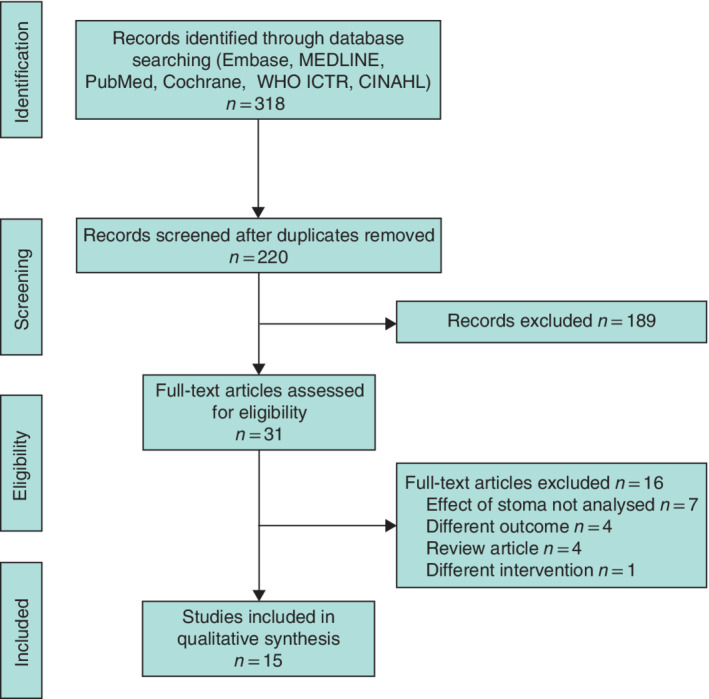
PRISMA diagram for the review

Some 220 studies were screened after deleting duplicates. After review of titles and abstracts to identify relevant studies, 31 manuscripts were assessed for inclusion. A final 15 studies^14^, ^15^, ^16^, ^17^, ^18^, ^19^, ^20^, ^21^, ^22^, ^23^, ^24^, ^25^, ^26^, ^27^, ^28^ were included (*Fig*. *1*), including 488 people with SCI who had been given an intestinal stoma. Eleven of these studies assessed QoL, 14 considered time spent on bowel care, and 12 assessed patient satisfaction with the stoma. Thirteen were retrospective cross‐sectional studies combining chart review and questionnaires or interviews. There were two case–control studies, one of which was prospective.

**Table 1:** Quality of life

Reference	No. of patients	Quality of life	Measure used
Cooper *et al*.^25^	26	QoL was improved in 76 per cent, unchanged in 24 per cent	Likert scale
Munck *et al*.^21^	23	QoL was improved in 50 per cent, unchanged in 40 per cent	Questionnaire
Luther *et al*.^24^	74 stoma, 296 controls	No difference in QoL	Questionnaire
Branagan *et al*.^18^	32	QoL was improved: 78 per cent ‘much better’ and 15 per cent ‘better’	Likert scale
Safadi *et al*.^16^	45	QoL was improved; QoL score improved from 60 to 80. This index was based on five general domains of QoL: physical health, psychosocial adjustment, body image, self‐efficacy and recreation/leisure	QoL index
De La Fuente *et al*.^19^	12	QoL was improved: 58 per cent ‘much better’ and 25 per cent ‘better’	Likert scale
Rosito *et al*.^23^	27	QoL was improved. QoL index significantly improved in the stoma group. Of five QoL domains assessed, there was a statistically significant increase in four: physical health, self‐efficacy, psychosocial status and recreation/leisure. Body image was unaffected	QoL index
Randell *et al*.^22^	52	No difference in QoL. Areas assessed included systemic symptoms, emotional function, social function, work function and bowel function	Questionnaire
Kelly *et al*.^20^	14	QoL was improved: 79 per cent ‘much better’ and 7 per cent ‘better’	Likert scale
Craven and Etchells^*26*^	17	QoL was improved. Exact figures not published, but authors commented that QoL was improved and patients consistently commented that they had more independence after stoma formation	Questionnaire
Stone *et al*.^17^	20	QoL was improved: 64 per cent ‘much better’, 27 per cent ‘better’ and 9 per cent unchanged	Likert scale

QoL, quality of life.

The quality assessment score for the 15 studies is shown in *Table S1* (supporting information). All studies had clearly defined objectives and study populations, all with good rates of follow‐up. However, these scores give some guidance regarding the source of study weakness. Some studies^14^, ^15^, ^16^ used validated scores to assess QoL, but others^17^, ^18^ relied instead on subjective questionnaires. This is often the case where outcomes are largely subjective, such as in this study. There were also several weaknesses inherent in the study design. For example, cross‐sectional studies are unable to analyse changes over time. None of the included studies employed power calculations.

### Quality of life

Eleven studies assessed directly whether QoL was improved following formation of an intestinal stoma (*Table* *1*). This was assessed in a variety of different ways; most commonly, patients were simply asked whether they thought their QoL was better or improved by stoma formation. Five studies^17^, ^18^, ^19^, ^20^, ^25^ used a five‐point Likert scale (much better, better, unchanged, worse, much worse). Four studies^21^, ^22^, ^24^, ^26^ used a questionnaire and two^16^, ^23^ used a QoL index made up of different components affecting QoL, including physical or systemic symptoms, psychosocial, body image, self‐efficacy, work function, recreation or leisure and bowel function.

**Table 2:** Time spent on bowel care

Reference	No. of patients	Time spent on bowel care
Cooper *et al*.^25^	26	Significantly reduced. Forty per cent spent over 1 h before stoma formation; this reduced to 0 per cent after stoma formation, with 88 per cent now taking 15 min or less
Bølling Hansen *et al*.^14^	18	Significantly reduced. Fifty per cent spent over 1 h before stoma formation; this reduced to 0 per cent after stoma formation, with over 70 per cent taking 15 min or less
Coggrave *et al*.^15^	92	Significantly reduced. Forty‐five per cent spent over 1 h before stoma formation; this reduced to 9 per cent, with 60 per cent now spending 15 min or less
Munck *et al*.^21^	23	Significantly reduced from 51 to 13 min per day
Branagan *et al*.^18^	32	Significantly reduced from 88 to 16 min per day
Safadi *et al*.^16^	45	Significantly reduced. In patients with a right‐sided colostomy the average time spent per day decreased from 102 to 11 min, for those with a left‐sided colostomy from 123 to 18 min, and for ileostomy from 73 to 13 min
De La Fuente *et al*.^19^	12	Significantly reduced from 70 to 21 min per day
Rosito *et al*.^23^	27	Significantly reduced from 117 to 13 min per day
Kelly *et al*.^20^	14	Significantly reduced from 75 to 12 min per day
Craven and Etchells^26^	17	All patients commented that they spent less time on bowel care. Exact figures not given
Saltzstein and Romano^27^	16	Significantly reduced from 95 to 34 min per day
Stone *et al*.^17^	20	Significantly reduced from 99 to 18 min per day
Frisbie *et al*.^28^	20	Significantly reduced, with the median time spent on bowel care reduced from 77 to 9 min per day

Nine^16^, ^17^, ^18^, ^19^, ^20^, ^21^, ^23^, ^25^, ^26^ of these 11 studies reported an improvement in QoL after stoma formation. The other two studies^22^, ^24^ showed no difference in QoL. In the studies using the Likert scale the results were strongly in favour of improved QoL with 93 per cent^18^, 83 per cent^19^, 86 per cent^20^, 91 per cent^17^ and 76 per cent^25^ of patients reporting either ‘much better’ or ‘better’ QoL following stoma formation. There was a greater chance of QoL being improved in patients whose stoma had been formed because of problems with their bowel, whereas stoma dissatisfaction was far more common when patients had not perceived bowel care as an issue before the surgery^17^.

One report^24^ used a questionnaire to compare seven areas assessing bowel‐related QoL in patients with a stoma with responses from a control group. Across all seven areas, the authors found no statistically significant differences between the two groups.

The only prospective study^22^ documented no difference in QoL. This study, however, compared patients with SCI who had had a stoma formed owing to significant bowel dysfunction after generally exhausting medical treatments with controls who were matched in terms of level of disability but not with regard to level of bowel dysfunction. These confounding baseline differences between the two groups may have led to them being merely equal after stoma surgery, perhaps explaining why there was no improvement in QoL in the stoma group.

### Time spent on bowel care

Time spent on bowel care was the most consistently assessed outcome, with 13 studies assessing this (*Table* *2*). All of these studies showed a substantial improvement in this measure. Importantly these studies highlighted just how long, on average, patients with SCI without a stoma generally have to spend each day on bowel care, with nine studies^14^, ^16^, ^17^, ^18^, ^19^, ^20^, ^23^, ^27^, ^28^ reporting that most patients had to spend more than 1 h on bowel care each day. Some patients were reported spending over 2 h each day on bowel care^16^. Eight studies^14^, ^15^, ^16^, ^20^, ^21^, ^23^, ^25^, ^28^ reported that, after stoma formation, time spent per day on bowel care for most patients was 15 min or less, with the longest time reported for bowel care being 34 min^27^.

**Table 3:** Satisfaction with stoma

Reference	No. of patients	Patient satisfaction
Cooper *et al*.^25^	26	Satisfaction was very high: 72 per cent scored at least 8 on a 10‐point Likert scale asking to rate how satisfied they were with their stoma; 96 per cent would recommend to a friend
Bølling Hansen *et al*.^14^	18	Only one patient wanted their stoma to be reversed; 66 per cent wished they had had it sooner
Coggrave *et al*.^15^	92	Over 82 per cent said they would have stoma surgery again, with 84 per cent saying they would recommend it to a friend; 16 per cent wanted stoma reversal
Branagan *et al*.^18^	32	All patients felt bowel care was easier; 78 per cent wished it had been done sooner
Safadi *et al*.^16^	45	Some 83–100 per cent of patients were satisfied with their stoma (depending on stoma location); 63–77 per cent wished it had been done sooner; 12 per cent or less would want their stoma reversed
De La Fuente *et al*.^19^	12	All patients had stomas created for management of pressure areas and not for bowel control; despite this, 75 per cent did not want their stoma reversed
Rosito *et al*.^23^	27	All patients (100 per cent) reported they were ‘satisfied’ with their stoma and 59 per cent were ‘very satisfied’; 70 per cent wished they had had it done sooner. All patients stated that the stoma improved independence
Kelly *et al*.^20^	14	All patients felt bowel care was easier, with 92 per cent wishing it had been done sooner. None wanted their stoma reversed, and 83 per cent said their independence had increased as a result of the stoma
Craven and Etchells^26^	17	Only one patient wanted their stoma closed, and this had been done to manage a colovesical fistula that had healed
Saltzstein and Romano^27^	16	Some 93 per cent reported satisfaction with their stoma and would recommend it to other patients. Only one patient wanted reversal
Stone *et al*.^17^	20	No patients in this study wanted their stoma reversed. Satisfaction was highest in group that had the stoma for gastrointestinal problems
Frisbie *et al*.^28^	20	No patient regretted stoma formation; all patients reported being happier with their bowel care

### Patient satisfaction with the stoma

All 12 studies that assessed patient satisfaction with their stoma reported high levels of satisfaction (*Table* *3*). Some studies^16^, ^23^, ^27^ that asked patients directly whether they were satisfied with the stoma reported high percentages giving a positive response, ranging between 83 and 100 per cent. Another report^25^ documented that 72 per cent of patients scored at least 8 when asked to score their satisfaction out of 10. Other studies reported patient satisfaction in a slightly more indirect manner; three studies^18^, ^20^, ^28^ reported all patients saying bowel care was now easier than before they had a stoma, and two^20^, ^23^ said independence was improved. Five studies^14^, ^16^, ^18^, ^20^, ^23^ asked patients whether they wished they had had a stoma sooner, and a high proportion, ranging from 63 to 92 per cent, responded positively to this question.

The rate of desire for stoma reversal was low in the reviewed studies, but some patients did want stoma reversal. This was generally a minority of patients: only one patient in three studies^14^, ^26^, ^27^, and between 12 and 16 per cent of patients in others^15^, ^16^. These patients were more likely to have been given inadequate information and counselling before surgery and to have had an ileostomy, and those more anxious about body image and odour were less likely to state they would be willing to undergo the surgery again^15^.

## Discussion

This review has shown that, in most patients with SCI, colostomy formation improves QoL, significantly reduces time spent on bowel care and increases independence; more than 90 per cent of these patients are satisfied with having a stoma.

SCI and bowel dysfunction can have a huge impact on QoL^6^. The majority of patients with SCI rank loss of bowel control as their greatest functional loss after mobility^7^, with half of these patients stating that bowel dysfunction negatively impacts their QoL^11^, ^12^. Therefore, obtaining good control of bowel function early will have a positive effect on these patients' QoL.

Bowel dysfunction is highly prevalent in patients with SCI and its causes are multifactorial. There are factors related intrinsically to the nature of the SCI, with a high‐level (cervical) injury or complete injury being associated with more severe bowel dysfunction^29^. Depending on the injury, the nerve supply to both the bowel itself and the sphincter and continence mechanisms are affected, commonly resulting in issues with both severe constipation and faecal incontinence. There are also causes extrinsic to the SCI that affect the bowel, such as pre‐existing conditions and co‐morbidities, reduced mobility, poly‐pharmacy and psychological disorders, particularly depression^30^. Some 62 per cent of patients with SCI report faecal incontinence as impacting negatively on their QoL, compared with 8 per cent of controls^31^. Bowel care also takes up a large proportion of patients' lives, with the majority needing to spend more than 1 h per day on bowel care alone^14^, ^16^, ^17^, ^18^, ^19^, ^20^, ^23^, ^27^, ^28^. In addition, bowel care can be physically challenging for carers and degrading for the patient.

Patients require pharmacological interventions, diet modification, anorectal stimulation, irrigation and abdominal massage to manage bowel function successfully, and sometimes surgery (including ostomy formation) is required. Due to stigma, colostomy formation is performed only once conservative management has ‘failed’. This process of trialling conservative measures first can take years, with patients often suffering a reduced QoL as a result of their poor bowel function for this period^15^, ^22^. Owing to the stigma associated with ostomy formation, many clinicians are reluctant to suggest the procedure early after a patient's injury. As an example, in 2012 the Multidisciplinary Association of Spinal Cord Injured Professionals released a 60‐page guideline for the management of neurogenic bowel dysfunction in individuals with central neurological conditions^32^; colostomy appears last on a list of 14 possible interventions, and only a small proportion of patients with SCI have a stoma^20^, ^33^.

Bowel dysfunction develops fairly rapidly after SCI^31^, ^34^, and colostomy formation within 12 months of SCI could be a safe and effective way of gaining independence and making bowel care easier^35^.

Nine of the 11 papers reporting on impact of QoL showed an improvement following stoma formation. This was especially true when patients had the stoma formed for management of bowel dysfunction. Low self‐efficacy has been shown^36^ to be associated with reduced QoL, and many patients reported that the stoma gave them increased independence^20^, ^23^. The second most common indication for stoma formation was for management of pressure areas; however, the majority of these patients still did not want the stoma reversed, even once pressure areas had healed^19^.

Looking at patients in the studies who were not happy with their stoma provides insight into who is more likely to have most benefit from a stoma. Patients who were not happy, or wanted the stoma reversed, were more likely to be those in whom the stoma had been formed for an indication other than bowel dysfunction^14^, ^23^, ^26^. Subgroups that had a stoma specifically for gastrointestinal problems all said that the stoma increased their QoL^17^. As for any patient receiving a stoma, adequate support and counselling before surgery are important, and lack of information is one of the reasons for concern, along with anxiety about appearance change or odour^29^.

It has also been shown^37^ that ensuring access to a specialist ostomy nurse in the first 3–6 months after stoma formation increases QoL. This is consistent with research into stomas in other (non‐SCI) populations with debilitating chronic bowel conditions, such as inflammatory bowel disease and faecal incontinence. These patients report high levels of satisfaction with their stoma^1^, ^38^, in contrast to some patients with cancer who are dissatisfied as the stoma was not seen to alleviate a complaint or symptom^39^.

In addition, the level and completeness of injury could also impact on which patients might benefit from a stoma. Cervical and complete injuries are associated with less manual dexterity and less independence, perhaps meaning that this group would derive greater benefit from a stoma than groups with a more distal or incomplete injury. One study^23^ compared outcomes in patients with a complete injury and those without, and found that, although QoL was improved significantly in both groups, there was no difference between them. The studies with a control group^22^, ^24^ ensured that levels of injury were matched between the two groups, but did not analyse these groups separately. The majority of the included studies^14^, ^15^, ^16^, ^17^, ^19^, ^20^, ^21^, ^22^, ^23^, ^25^, ^26^, ^27^, ^28^ reported levels of injury in their patients, but again did not analyse these groups separately. Stoma formation in patients with SCI is as safe as that in patients without SCI, with comparable complication rates^40^; however, complications will, of course, still occur. The most common complications reported were rectal discharge, stoma prolapse, wound healing issues and skin irritation^15^, ^17^, ^18^, ^20^, ^26^. Several patients across the studies even required further surgery to remove the remaining colon, to treat the diversion colitis and rectal discharge or to repair the parastomal hernia. Most importantly, despite the presence of these complications, overall stoma formation still translated into an improved QoL. It has also been shown^23^ that colostomy formation significantly reduces the number of hospitalizations for bowel dysfunction.

The location and type of stoma is also important. Colostomy formation was recommended over ileostomy formation^15^, ^20^, ^21^, ^28^.

The main limitations of this review are that most of the included studies were retrospective and only small numbers of patients were involved, so the quality of evidence was modest. In addition, there was heterogeneity in the types of spinal injury, in the tools used to assess QoL, and in time from stoma formation. Despite this, the results were consistent across most of the studies. Further prospective research in this area would be useful.

## Disclosure

The authors declare no conflict of interest.

## Supporting information


**Appendix S1**: Supporting information
