# Supplementary material for: Colostomy and quality of life after spinal cord injury: systematic review
Source: BJS Open. 2020 Aug 27;4(6):1054–61. doi: 10.1002/bjs5.50339 (PMC7709367; doi:10.1002/bjs5.50339)
Supplement: Supplementary file 1 — Appendix S1: Supporting information [file BJS5-4-1054-s001.docx]

**BJS5_50339**

**Colostomy and quality of life after spinal cord injury: systematic review**

**O. Waddell, A. McCombie and F. Frizelle**

**Table S1** Studies included in the systematic review

| **Title** | **Year** | **Author** | **n** | **Design** | **Country** | **quality assessment score** |
| --- | --- | --- | --- | --- | --- | --- |
| Outcomes following stoma formation in patients with spinal cord injury | 2019 | Cooper et al | 26 | Retrospective Cross-sectional study | Australia | 8 of 9 |
| Bowel function and quality of life after colostomy in individuals with spinal cord injury. | 2016 | Bolling Hansen R et al | 18 | Retrospective Cross-sectional study | Denmark | 6 of 9 |
| The impact of stoma for bowel management after spinal cord injury. | 2012 | Coggrave M.J. et al | 92 | Multi-centre retrospective cross-sectional survey | UK | 5 of 9 |
| Intestinal stoma in patients with spinal cord injury: A retrospective study of 23 patients. | 2008 | Munck J. et al | 23 | Retrospective Cross-sectional study | Belgium | 5 of 10 |
| A comparison of patient outcomes and quality of life in persons with neurogenic bowel: Standard bowel care program vs colostomy. | 2005 | Luther S.L. et al | 74 with stoma, 296 controls | Retrospective Case-Control study | USA | 10 of 12 |
| Effect of stoma formation on bowel care and quality of life in patients with spinal cord injury. | 2003 | Branagan G. et al | 32 | Retrospective Cross-sectional study | UK | 4 of 9 |
| Which stoma works better for colonic dysmotility in the spinal cord injured patient?. | 2003 | Safadi B.Y. et al | 45 | Retrospective Cross-sectional study | USA | 4 of 9 |
| Elective Stoma Construction Improves Outcomes in Medically Intractable Pressure Ulcers. | 2003 | De La Fuente S.G. et al | 12 | Retrospective Cross-sectional study | USA | 3 of 9 |
| The effects of colostomy on the quality of life in patients with spinal cord injury: A retrospective analysis. | 2002 | Rosito O. et al | 27 | Retrospective Cross-sectional study | USA | 7 of 10 |
| Does a colostomy alter quality of life in patients with spinal cord injury? A controlled study. | 2001 | Randell N. et al | 52 | Prospective Case-Control study. | New Zealand | 9 of 10 |
| The role of intestinal stoma in patients with spinal cord injury. | 1999 | Kelly S.R. et al | 14 | Retrospective Cross-sectional study | UK | 5 of 10 |
| A review of the outcome of stoma surgery on spinal cord injured patients. | 1998 | Craven ML et al | 17 | Retrospective Cross-sectional study | Australia | 5 of 10 |
| The efficacy of colostomy as a bowel management alternative in selected spinal cord injury patients. | 1990 | Saltzstein R.J. et al | 16 | Retrospective Cross-sectional study | USA | 4 of 9 |
| Colostomy as treatment for complications of spinal cord injury. | 1990 | Stone J.M. at al | 20 | Retrospective Cross-sectional study | USA | 5 of 10 |
| Effect of enterostomy on quality of life in spinal cord injury patients. | 1986 | Frisbie J.H. et al | 20 | Retrospective Cross-sectional study | USA | 5 of 10 |
